# Supplementary material for: Modeling glioblastoma heterogeneity as a dynamic network of cell states
Source: Mol Syst Biol. 2021 Sep 16;17(9):e10105. doi: 10.15252/msb.202010105 (PMC8444284; doi:10.15252/msb.202010105)
Supplement: Supplementary file 5 — Source Data for Figure 3 [file MSB-17-e10105-s001.zip › Figure3A_sourcedata/GSEA_3065/nosigdb_state3.GseaPreranked.1622718426028/IVY_IT.html]

Details for gene set IVY\_IT[GSEA]

|  || Dataset | state3 |
| Phenotype | NoPhenotypeAvailable |
| Upregulated in class | na\_neg |
| GeneSet | IVY\_IT |
| Enrichment Score (ES) | -0.5469095 |
| Normalized Enrichment Score (NES) | -1.5884912 |
| Nominal p-value | 0.0068728523 |
| FDR q-value | 0.03292566 |
| FWER p-Value | 0.521 |
Table: GSEA Results Summary

  

Fig 1: Enrichment plot: IVY\_IT      
 Profile of the Running ES Score & Positions of GeneSet Members on the Rank Ordered List

  

| PROBE | GENE SYMBOL | GENE\_TITLE | RANK IN GENE LIST | RANK METRIC SCORE | RUNNING ES | CORE ENRICHMENT || 1 | DNM1 |  |  | 310 | 0.240 | 0.0205 | No |
| 2 | ATP1B1 |  |  | 1102 | 0.129 | -0.0400 | No |
| 3 | APOD |  |  | 1113 | 0.129 | -0.0111 | No |
| 4 | KIF1A |  |  | 1583 | 0.099 | -0.0419 | No |
| 5 | OLFM1 |  |  | 1809 | 0.086 | -0.0476 | No |
| 6 | PLP1 |  |  | 2047 | 0.074 | -0.0575 | No |
| 7 | GAD1 |  |  | 2334 | 0.063 | -0.0756 | No |
| 8 | PAK3 |  |  | 3132 | 0.038 | -0.1582 | No |
| 9 | APLP1 |  |  | 3403 | 0.030 | -0.1820 | No |
| 10 | FBXL16 |  |  | 4345 | 0.005 | -0.2887 | No |
| 11 | CCK |  |  | 4463 | 0.002 | -0.3016 | No |
| 12 | KCNJ16 |  |  | 5242 | -0.017 | -0.3869 | No |
| 13 | ZNF365 |  |  | 5473 | -0.023 | -0.4078 | No |
| 14 | METTL7B |  |  | 6482 | -0.056 | -0.5104 | No |
| 15 | PRKCZ |  |  | 6567 | -0.059 | -0.5062 | No |
| 16 | NDRG4 |  |  | 6923 | -0.077 | -0.5290 | Yes |
| 17 | SLC25A18 |  |  | 6927 | -0.077 | -0.5113 | Yes |
| 18 | NRGN |  |  | 7043 | -0.084 | -0.5049 | Yes |
| 19 | STXBP1 |  |  | 7303 | -0.101 | -0.5111 | Yes |
| 20 | PLEKHB1 |  |  | 7359 | -0.105 | -0.4930 | Yes |
| 21 | SEPT4 |  |  | 7508 | -0.118 | -0.4825 | Yes |
| 22 | GABBR2 |  |  | 7511 | -0.118 | -0.4551 | Yes |
| 23 | CHN1 |  |  | 7653 | -0.133 | -0.4403 | Yes |
| 24 | ENHO |  |  | 8124 | -0.210 | -0.4453 | Yes |
| 25 | NEFL |  |  | 8203 | -0.230 | -0.4007 | Yes |
| 26 | UCHL1 |  |  | 8299 | -0.258 | -0.3515 | Yes |
| 27 | PCP4 |  |  | 8402 | -0.297 | -0.2940 | Yes |
| 28 | BEX1 |  |  | 8450 | -0.314 | -0.2261 | Yes |
| 29 | TAGLN3 |  |  | 8479 | -0.330 | -0.1523 | Yes |
| 30 | S100B |  |  | 8562 | -0.373 | -0.0747 | Yes |
| 31 | GFAP |  |  | 8610 | -0.413 | 0.0162 | Yes |
Table: GSEA details [plain text format]

  

Fig 2: IVY\_IT: Random ES distribution      
 Gene set null distribution of ES for **IVY\_IT**
